# Ecological diversity and co-occurrence patterns of bacterial community through soil profile in response to long-term switchgrass cultivation

**DOI:** 10.1038/s41598-017-03778-7

**Published:** 2017-06-15

**Authors:** Shubin He, Lixiang Guo, Mengying Niu, Fuhong Miao, Shuo Jiao, Tianming Hu, Mingxiu Long

**Affiliations:** 1 College of Animal Science and Technology, Northwest A&F University, Yangling, Shaanxi 712100 P.R. China; 2 College of Animal Science and Technology, Qingdao Agriculture University, Qingdao, Shandong 266109 China; 3 State Key Laboratory of Crop Stress Biology in Arid Areas, College of Life Sciences, Northwest A&F University, Yangling, Shaanxi 712100 P. R. China

## Abstract

Switchgrass (*Panicum virgatum* L.) is a cellulosic biofuel feedstock and their effects on bacterial communities in deep soils remain poorly understood. To reveal the responses of bacterial communities to long-term switchgrass cultivation through the soil profile, we examined the shift of soil microbial communities with depth profiles of 0–60 cm in five-year switchgrass cultivation and fallow plots. The Illumina sequencing of the 16S rRNA gene showed that switchgrass cultivation significantly increased microbial OTU richness, rather than microbial Shannon diversity; however, there was no significant difference in the structure of microbial communities between switchgrass cultivation and fallow soils. Both switchgrass cultivation and fallow soils exhibited significant negative vertical spatial decay of microbial similarity, indicating that more vertical depth distant soils had more dissimilar communities. Specifically, switchgrass cultivation soils showed more beta-diversity variations across soil depth profile. Through network analysis, more connections and closer relationships of microbial taxa were observed in soils under switchgrass cultivation, suggesting that microbial co-occurrence patterns were substantially influenced by switchgrass cultivation. Overall, our study suggested that five-year switchgrass cultivation could generated more beta-diversity variations across soil depth and more complex inter-relationships of microbial taxa, although did not significantly shape the structure of soil microbial community.

## Introduction

Switchgrass (*Panicum virgatum* L.) is a perennial C-4 grass with high photosynthetic efficiency and biomass production potential^1^. It has received considerable attentions during the last several decades since it is recognized as a promising crop for biofuel production by the US Department of Energy (DOE) Herbaceous Energy Crops Program (HECP)^2–4^. The widespread of this perennial biofuel crops could shift the land use towards the renewable, biomass-based energy systems, and influence the soil ecosystems subsequently^5^. Particularly, soil microbes can respond rapidly to the environmental changes caused by plant^6, 7^. The plant could regulate soil microbial community structure via root exudates^8^. Switchgrass can release up to 20% of fixed carbon to the rhizosphere through exudation^9^. Although agronomic knowledge of switchgrass has grown increasingly^2, 4^, however, their influence on soil microbial community still remain uncovered.

Soil microbes play fundamental roles in soil biogeochemical processes of the carbon, nitrogen, and inorganic element cycles^10^. The vast majority of researches on soil microbial communities have focused on the top 15 cm of the soil column or less, therefore our understanding of soil microbes is limited to surface horizons^11, 12^. The microbial biomass often exhibits exponential decreases with depth and is greatest in surface soil, while there is still a large population of microbes in the subsoil (below 15 cm) because of the large volume throughout the depth of soil profile^11–14^. Due to the proximity to parent material, the deeper microbes might play potential important roles in soil formation processes, ecosystem biochemistry and pollutant degradation, as well as in maintaining the quality of groundwater^15–17^. The sub-surface horizons can harbor a large amount of organic C during long-term turnover, associating with the activity of microbes in subsoil^18, 19^. Previous studies have showed that microbial communities could significantly change with soil depths, and the microbial diversity of microorganisms typically decreases with depth^11, 20^. However, the characterization and spatial variability of microbial communities at deeper soils remains poorly understood under the influence of Switchgrass growing.

Microorganisms form complex interaction webs within a specific ecological niche, and understanding the interactions among microorganisms is important to explore the complexity of functional processes^21^. Co-occurrence network analysis could provide comprehensive perspective into the complex microbial interactions, such as commensalism, competition and predation^22^. However, most of the previous studies on the microbial community structures have been conducted based on the technologies of clone library analysis, community-level physiological profiles, phospholipid fatty acids (PLFAs), terminal restriction fragment length polymorphism (T-RFLP) and denaturing gradient gel electrophoresis (DGGE). These methods provided limited information to comprehensively resolve the phylogenetic responses of microbial communities to environmental changes due to the small number of sequences analyzed^23^. Recently the next-generation sequencing technologies have made broad and deep surveys of microbial communities taking full advantage of the network analysis approaches^24^. It could grab rare species and describe the overall microbial community diversity^25^. Therefore, network analysis have been applied to explore the microbial co-occurrence patterns in diverse environments including marine water^26^, soil^22, 27^ and activated sludge^28^. While, we still have only a limited understanding of the effects of Switchgrass growing on the co-occurrence patterns of microbial communities.

In present study, we conducted a metagenomic analysis of soil microbial communities via high-throughput sequencing of the 16S rRNA gene to examine their shifts with depth profiles and switchgrass cultivation. Soil samples were obtained from a five-year switchgrass experimental area in the Guanzhong plain of Shaanxi Province. The objective was to investigate the responses of bacterial communities to long-term switchgrass cultivation within the soil profile. Specifically, we addressed the following detailed questions: (i) Do switchgrass cultivations change the structure of bacterial community throughout the depth of soil profile? (ii) How the bacterial communities shift with the soil depths? (iii) What are the co-occurrence patterns between bacterial taxa responding to switchgrass cultivation?

## Results

### Distribution of bacterial taxa

**Figure 1:**
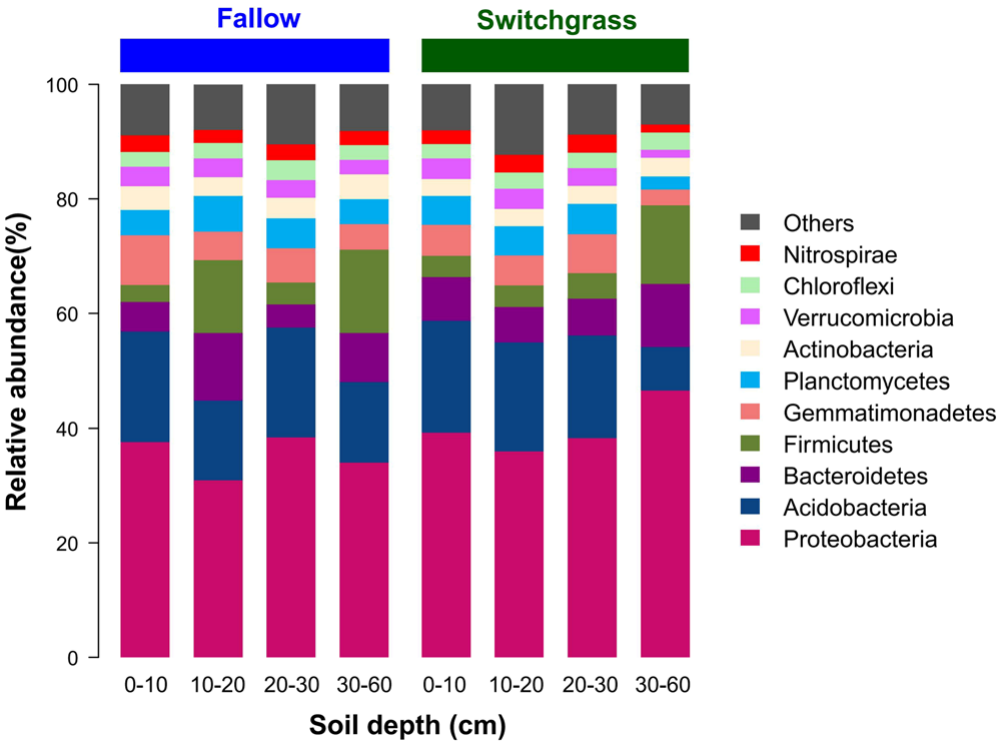
Relative abundances of bacterial phyla at 0–60 cm depth in switchgrass and fallow soils.

A data set of 1,116,764 quality sequences was produced from the soil samples in switchgrass cultivation and fallow plots with four soil depth profiles (Table 1), including 0–10 cm (Layer 1), 10–20 cm (Layer 2), 20–30 cm (Layer 3) and 30–60 cm (Layer 4). The mean number of sequences per sample (n = 24) was 46,532 (max = 58,071, min = 29,126, SD = 8941). The total OTU number was 12,547 defined by 97% sequence similarity. These OTUs were assigned to 64 phyla, 171 classes, 269 orders, 336 families and 526 genera. Of the OTUs, 99.5% (12,489 OTUs) were Bacteria and only 0.4% (54 OTUs) were affiliated with the Archaea domain. Five bacterial phyla including Proteobacteria, Acidobacteria, Bacteroidetes, Firmicutes and Gemmatimonadetes were predominant (relative abundance >5%), and accounted for 74.5% of the total sequences; and in addition, Planctomycetes, Actinobacteria, Verrucomicrobia, Chlorobi and Nitrospirae were present in most soil samples at low relative abundances (Fig. 1). At class level, Gammaproteobacteria (13.8%), Alphaproteobacteria (9.3%), Betaproteobacteria (6.7%) and Deltaproteobacteria (6.3%) were the dominant Proteobacteria with an overall relative abundance of 37.6%; Acidobacteria-6, Clostridia, Bacteroidia and Chloracidobacteria were also predominant groups, accounted for 22.0% of the total sequences in the soil samples (Supplementary Fig. S1). In general, Archaea accounted for 2.1% of all the sequences, and the the dominant taxa was *Candidatus Nitrososphaera* (phylum Crenarchaeota).

**Table 1:** Microbial alpha-diversity characteristics for the fallow and switchgrass soils in four layers.

Soil samples		No. of high quality reads		Diversity Index							
				Observed OTU_97_		Chao1		Shannon		Coverage	
		Average	SD*	Average	SD	Average	SD	Average	SD	Average	SD
Fallow	Layer 1	48925	7455	3786	770	5038.84	1423.12	10.11	0.44	0.95	0.02
	Layer 2	46956	10201	3480	857	4696.74	1529.37	9.64	0.99	0.96	0.02
	Layer 3	43865	12645	3802	369	4924.32	1293.83	10.26	0.05	0.96	0.02
	Layer 4	35819	5319	3005	413	3459.12	460.01	9.31	0.71	0.97	0.00
Switchgrass	Layer 1	51365	8028	4307	192	5866.10	653.67	10.41	0.15	0.95	0.01
	Layer 2	45093	13879	4018	871	5686.97	1263.94	10.09	0.72	0.95	0.01
	Layer 3	47804	4410	4101	275	5538.59	451.94	10.35	0.14	0.95	0.01
	Layer 4	52428	3250	3590	565	5050.41	678.51	8.69	1.35	0.95	0.01

*Standard deviation.

### Assembly patterns of bacterial community

**Figure 2:**
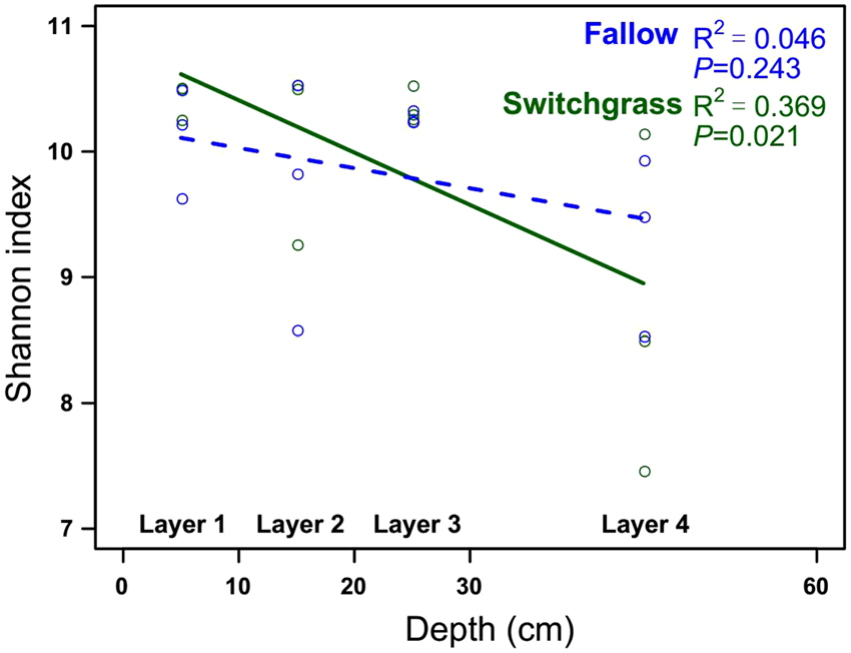
Changes in microbial diversity (Shannon index) with soil depth in switchgrass and fallow plots, estimated via linear regression.

Microbial alpha-diversity was measured using the observed OTU richness and Shannon–Wiener index. The observed OTU richness was significantly higher in switchgrass cultivation soil samples than that in fallow soils, tested by Wilcoxon rank-sum test (*P* < 0.05; Supplementary Fig. S2). While, Shannon index did not significantly different between these two groups. On the other hand, we found Shannon index significantly decreased with soil depth in switchgrass cultivation plots (*P* < 0.05); whereas, this trend was not significant in fallow plots (Fig. 2). And the observed OTUs richness did not show significantly change with soil depth in either switchgrass cultivation or fallow plots.

**Figure 3:**
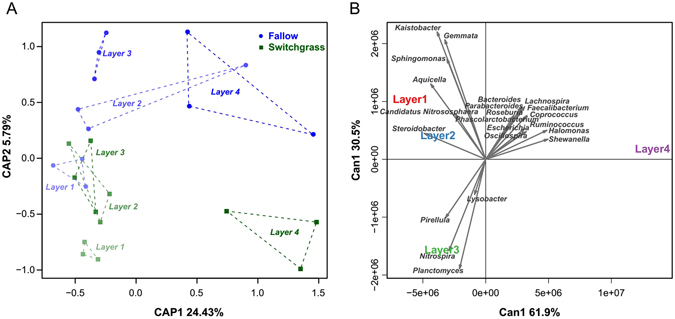
Microbial distribution patterns varied with soil depth in switchgrass and fallow plots. (**A**) Constrained analysis of the principal coordinates (CAP) of microbial communities following four soil layers of different plots (Layer 1: 0–10 cm, Layer 2: 10–20 cm, Layer 3: 20–30 cm and Layer 4: 30–60 cm) based on the Bray–Curtis distance. (**B**) Canonical discriminant analysis (CDA) comparing soil layers against microbial taxa loadings based on genera with relative abundance levels >0.5%. Arrows represent the degree of correlation between each taxon and each layer as a measure of the predictive discrimination of each layer.

The CAP analysis based on Bray–Curtis distance (Fig. 3A), demonstrated that bacterial community varied with depth, which were confirmed by ANOSIM (*P* < 0.05). The Canonical discriminant analysis (CDA) of the predominant microbial taxa (relative abundance >0.5%) at genus levels revealed taxonomic associations with soil depth (Fig. 3B). Different layers of soil profiles distinguished specific microbial taxa. In layer 1, *Aquicella*, *Kaistobacter*, *Sphingomonas* and *Gemmata* were the abundant genera; *Steroidobacter* and *Candidatus Nitrososphaera* were dominant in soils of layer 2; *Lysobacter*, *Pirellula*, *Nitrospira* and *Planctomyces* were dominant in layer 3; *Halomonas*, *Shewanella and Ruminococcus* were abundant genera in soils of layer 4.

There was no significant difference in the structure of microbial communities between switchgrass cultivation and fallow soils, either in integrate soil profiles (ANOSIM *P* = 0.113; PERMANOVA *P* = 0.203) or each single layer. While some significant taxonomic differences between these two groups soils were examined by Wilcoxon rank-sum test (*P* < 0.05) based on the top 1000 most abundant OTUs (Supplementary Figs S3 and S4). For example, *Novosphingobium*, *Fluviicola*, *Flavobacterium*, *Alcanivorax*, *Shewanella* and *Sorangium* were significantly higher in relative abundance in soils with switchgrass cultivation; whereas, the abundance of families Rhodospirillaceae and Gaiellaceae, and the genera *Gemmata* and *Pilimelia* increased significantly in fallow soils.

### Vertical spatial variations of bacterial community

**Figure 4:**
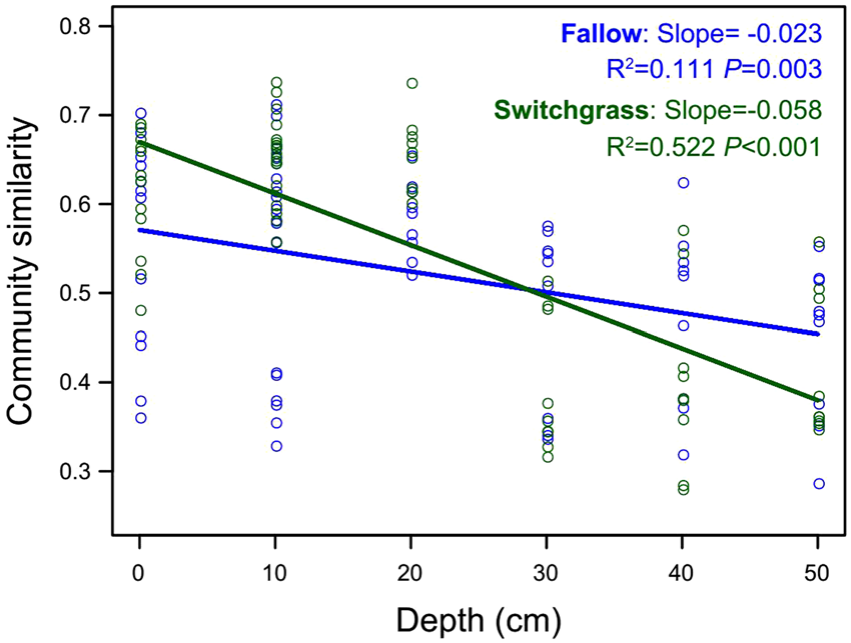
The vertical spatial variations of bacterial community similarity in switchgrass and fallow soils, estimated the relationships between soil depth profiles and microbial community similarities based on Bray–Curtis distance. The lines denote the least-squares linear regressions across soil depth.

To investigate the vertical spatial variations of bacterial community, we estimated the relationships between soil depth profiles and bacterial community similarities based on Bray–Curtis distance (Fig. 4). The significant negative vertical spatial decay of bacterial community relationships were found in linear regression for both switchgrass cultivation and fallow soils, indicating that more vertical depth distant soils had more dissimilar communities. In particular, the switchgrass cultivation soils had a steeper slope, indicating that there were more beta-diversity variations with the increased vertical depth under switchgrass cultivation.

To further explore vertical spatial variations of the dominated bacterial taxa, we estimated the correlations between relative abundance of these taxa and soil depths via Pearson coefficient (Supplementary Table S1 and Table 2). In fallow soils, phylum Crenarchaeota and classes Gemmatimonadetes, Thaumarchaeota, Saprospirae and Cytophagia were significantly and negatively correlated with soil depth; and classes Gammaproteobacteria were positively correlated with soil depth. For switchgrass cultivation soils, as soil depth increased, the relative abundances of phyla Acidobacteria, Verrucomicrobia and Armatimonadetes were significantly decreased in switchgrass cultivation soils; while, the abundance of Firmicutes and Cyanobacteria significantly increased. At class level, Gammaproteobacteria, Clostridia and Bacteroidia were significantly and positively correlated with soil depth; and Acidobacteria-6, Betaproteobacteria, Chloracidobacteria, Pedosphaerae, Cytophagia and Saprospirae were negatively correlated with soil depth. Additionally, the significant taxa were more in switchgrass cultivation soils than in fallow soils, confirmed more beta-diversity variations under switchgrass cultivation.

**Table 2:** The vertical spatial variations of the dominated microbial taxa at class level in the fallow and switchgrass soils, correlations between relative abundance of these taxa and soil depths were estimated via Pearson coefficient.

Class	Fallow		Switchgrass	
	Pearson coefficient	*P* value	Pearson coefficient	*P* value
Gammaproteobacteria	**0.598**	**0.040**	**0.603**	**0.038**
Alphaproteobacteria	−0.372	0.233	−0.393	0.207
Acidobacteria-6	−0.186	0.562	**−0.747**	**0.005**
Betaproteobacteria	−0.541	0.069	**−0.627**	**0.029**
Clostridia	0.272	0.393	**0.632**	**0.028**
Deltaproteobacteria	−0.505	0.094	−0.476	0.118
Bacteroidia	0.197	0.539	**0.593**	**0.042**
Chloracidobacteria	−0.264	0.407	**−0.699**	**0.011**
Planctomycetia	−0.057	0.861	−0.490	0.105
Nitrospira	−0.085	0.792	−0.384	0.218
Gemmatimonadetes	**−0.678**	**0.015**	−0.468	0.125
Pedosphaerae	−0.269	0.398	**−0.636**	**0.026**
Thaumarchaeota	**−0.708**	**0.010**	−0.271	0.394
Cytophagia	**−0.629**	**0.028**	**−0.671**	**0.017**
PRR-12	0.401	0.196	−0.331	0.294
Saprospirae	**−0.757**	**0.004**	**−0.767**	**0.004**
Gemm-1	0.411	0.184	**−**0.084	0.794
Anaerolineae	0.101	0.755	0.255	0.423
iii1-8	**−0.728**	**0.007**	**−**0.494	0.102
Actinobacteria	0.389	0.212	0.536	0.073

### Co-occurrence network analysis

**Figure 5:**
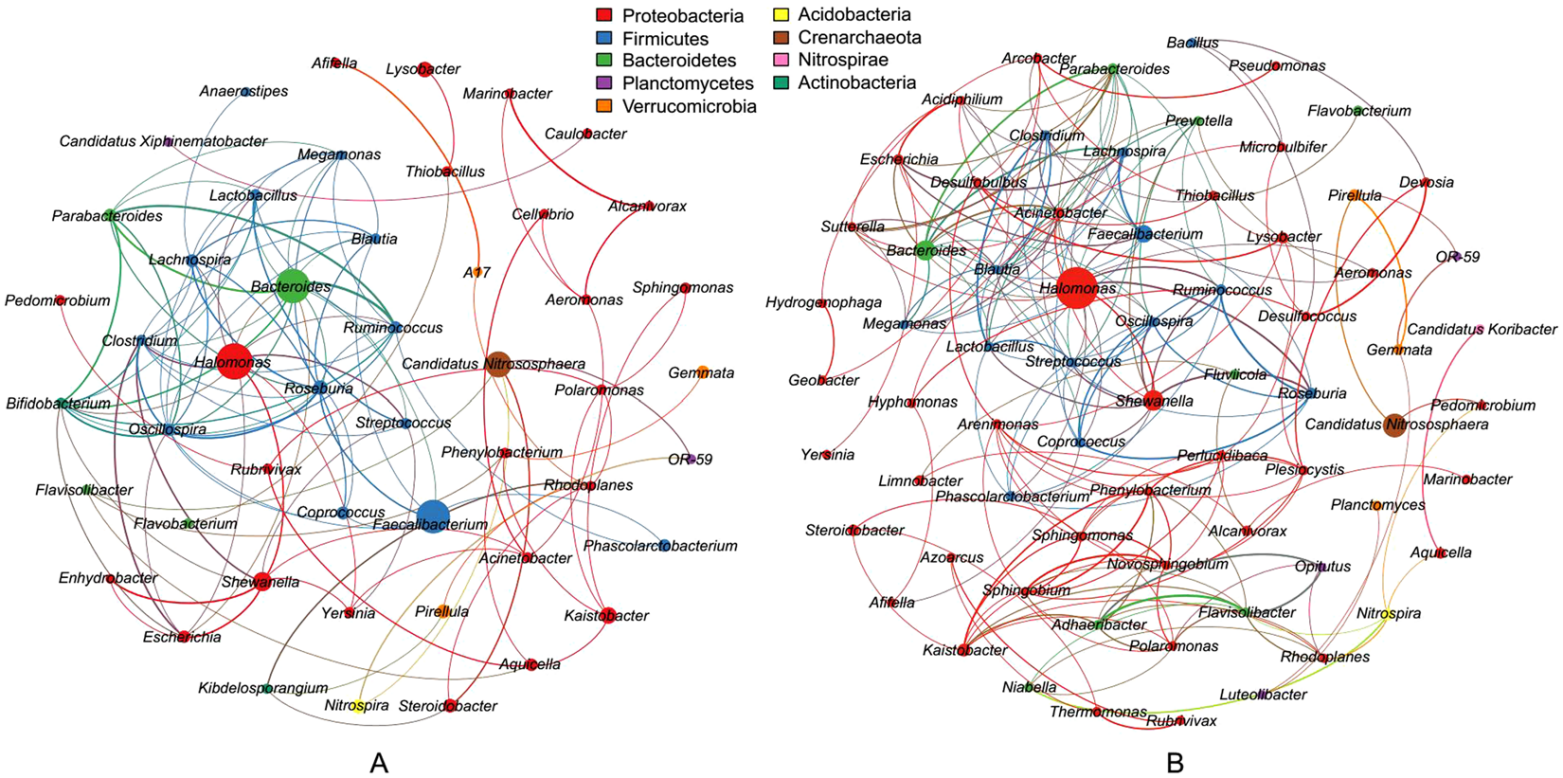
Network of co-occurring microbial genera based on correlation analysis for fallow (**A**) and switchgrass cultivation (**B**) soils. A connection stands for a strong (Spearman’s ρ > 0.6) and significant (*P* < 0.01) correlation. The size of each node is proportional to the relative abundance; the thickness of each connection between two nodes (edge) is proportional to the value of Spearman’s correlation coefficients. The nodes were colored by phylum.

The soil microbial networks were generated for switchgrass cultivation and fallow soils, respectively (Fig. 5). The topological properties were calculated to describe the complex pattern of inter-relationships among nodes, and to distinguish differences in taxa correlations between these two group soils (Table 3). Specifically, the structural properties of the switchgrass network were greater than the fallow network, indicating more connection and closer relationships of microbial taxa under switchgrass cultivation.

**Table 3:** Topological properties of co-occurring networks obtained from switchgrass cultivation and fallow soils.

	Nodes	Edges	Modularity (MD)	Clustering coefficient (CC)	Average path length (APL)	Network diameter (ND)	Average degree (AD)
Fallow	49	113	0.456	0.565	2.608	4.764	2.306
Switchgrass	69	204	0.577	0.592	5.081	11.537	2.957

Based on betweenness centrality scores, the top five genera identified as keystone taxa were *Arenimonas*, *Clostridium*, *Thiobacillus*, *Lysobacter* and *Nitrospira* in fallow network; *Shewanella*, *Acinetobacter*, *Rhodoplanes*, *Aeromonas* and *Bacteroides* were keystone taxa in switchgrass network. The keystone taxa differed greatly between these two networks. Furthermore, betweenness centrality of the switchgrass network was much stronger than that of the fallow network (*P* < 0.05, Wilcoxon rank-sum test; Supplementary Fig. S5), which could confirm that switchgrass network have more complex inter-relationships of microbial taxa.

## Discussion

As a high photosynthetic efficiency and biofuel production potential perennial C-4 grass, the widespread planting of switchgrass might provide great economic value. However, whether switchgrass cultivation influences the soil ecosystems, particularly in the deep soils profiles, still remain uncovered. The present study aimed to reveal the responses of microbial communities to long-term switchgrass cultivation within the soil profiles of 0–60 cm. Our results showed that switchgrass cultivations did not significantly change the structure of soil microbial community, but generated more beta-diversity variations across soil depth and more complex inter-relationships of microbial taxa.

Plant could regulate soil microbial community structure through the root architecture, exudates, and mucilage^8^. The rhizodeposits from plant roots appear to be a major driving force in the regulation of microbial diversity and activity^29–31^. Previous study revealed that switchgrass could enrich specific microbial species in the rhizosphere, which were able to utilize root exudates^5^. However, we did not observe significant difference in bacterial communities between switchgrass cultivation and fallow soils. It might be explained that the affected zones of roots are small, and plants might not be enough to influence the whole soil ecosystems. In our study, the soils were obtained from a five-year switchgrass cultivation area. Thus, our results suggest that long-term switchgrass cultivations could not significantly change the structure of soil microbial communities. In other context, switchgrass cultivation caused some specific taxonomic differences compared with the fallow soils. The enriched taxa in soils with switchgrass cultivation were mainly affiliated with Proteobacteria, Bacteroidetes and Acidobacteria (Supplementary Fig. S3). Previous study reported that Proteobacteria and Acidobacteria were the dominant members in the switchgrass rhizosphere soils^5^. Particularly, Proteobacteria were active utilizers of fresh photosynthate; while, Acidobacteria preferred to complex organic matter, rather than simple root-derived dissolved organic carbon^5^. Soil Bacteroidetes were typically copiotrophic and were most abundant in nutrient rich soils, including rhizosphere soils^32^. Additionally, we found that genera *Novosphingobium*, *Fluviicola* and *Flavobacterium* were enriched under switchgrass cultivation. *Novosphingobium* and *Flavobacterium* were the dominant root exudate utilizers in switchgrass rhizosphere reportedly^32^. *Fluviicola* was isolated as an endophytic bacterium through addition of plant extract to nutrient media^33^. Root exudates are a key factor in shaping microbiome, and the ability to utilize root exudates is an important trait that allows microorganisms to be competitive in the rhizosphere^5^. Additionally, soil microbiome–plant feedback mechanisms are closely associated with ecosystem function and primary productivity in terrestrial habitats^34, 35^. Switchgrass has been reported to require much less fertilizer input and to generate high yields compared to many other crops^36, 37^. The enriched microbial taxa in switchgrass cultivation soils were selectively assembled, and might be benefit of plant growth and health. These beneficial microbes might support nutrients for the high annual biomass production of switchgrass, which usually referred to as plant growth promoting rhizobacteria (PGPR). In our study, the switchgrass cultivation enriched bacteria belonged to *Flavobacterium*, Xanthomonadaceae and Pseudomonadaceae were reported as PGPR^38, 39^.

Previous work demonstrated that the diversity of microorganisms typically decreases with soil depth^12, 14^. In present study, we only found that microbial Shannon diversity significantly decreased with soil depth in switchgrass cultivation plots, while not in fallow plots. Switchgrass cultivation might provide nutrients via root exudates, which might different across the soil profiles due to the length of root. This could be supported by another work of our lab, which was conducted in the same experimental area (manuscript submitted). My colleagues found that soil organic carbon was found significantly higher in switchgrass cultivation soils than that in fallow soils through soil layers (Supplementary Fig. S6). This could also explain that the microbial richness was significantly higher under switchgrass cultivation.

Soil depth had a highly significant effect on the structure of microbial communities, especially in the switchgrass cultivation plots. Both switchgrass cultivation and fallow soils exhibited significant negative vertical spatial decay of microbial community similarity relationships, and the switchgrass cultivation soils had a steeper slope (Fig. 4). This indicated that more vertical depth distant soils had more dissimilar communities, and switchgrass cultivation generated more beta-diversity variations across soil depth. Switchgrass cultivation could provide different kinds of nutrients via root exudates, resulting in the complex environmental heterogeneity throughout the soil depth. Higher amplitude of variation in environmental conditions could explain the high variations in beta-diversity^40^. Previous researches showed that the subsoil microbial communities were distinct from topsoil communities^11, 12, 17^. In present study, the relative abundance of Firmicutes, Cyanobacteria, Gammaproteobacteria and Bacteroidia increased with soil depth. Some observed changes was similar to other studies^11, 20, 41^. Firmicutes can survive in extreme environments, and Cyanobacteria generally occur in harsh desert environments^42^. Gammaproteobacteria were likely to promote plant and root growth by fixing nitrogen and producing growth hormones^43^. On the other hand, the relative abundance of Acidobacteria, Verrucomicrobia, Crenarchaeota, Betaproteobacteria and Gemmatimonadetes decreased as soil depth increases. Previous works reported that Acidobacteria was negatively correlated with pH, which was increased with soil depths^14, 44, 45^. Crenarchaeota, dominated by class Thaumarchaeota in our study, is widespread speculation of driving the autotrophic nitrification^46^. Soil Verrucomicrobia were oligotrophic and able to grow under conditions of low C availability^47^. While ecological niches inhabited by Crenarchaeota and Verrucomicrobia remain largely undetermined^12^.

Although the entire soil microbial communities were not significantly changed, the microbial inter-relationships were substantially influenced by switchgrass cultivation. Through co-occurrence network analysis, we found that structural properties of the switchgrass network were greater than the fallow network, indicating more connection and closer relationships of microbial taxa under switchgrass cultivation. Comparing network-level topological features can provide us with insight into variations in the co-occurrence patterns between different communities^48^. Additionally, betweenness centrality of the switchgrass network was much stronger than that of the fallow network, which could confirmed more complex inter-relationships of microbial taxa under switchgrass cultivation. Discerning the modules maintaining the connectivity in network, betweenness centrality represents the potential of an individual node influence on the interactions of other nodes in the network, and has been used to define the keystone species in the ecosystems^49–52^. High betweenness centrality value indicates a core and central location of this node in the network, whereas low betweenness centrality value indicates a more peripheral location^48^. Switchgrass could secrete root exudates to the soil ecosystems, including sugars, amino acids and other organic acids^53^, which can be easily utilized by complex microbial communities. This might be supported by higher values of soil organic carbon under switchgrass cultivation (Supplementary Fig. S6). For microorganisms, wide niches can support the coexistence of species within the communities^54^. In this case, plants could supply carbon (C) to soil generating intense microbial activities and interactions^55^. In previous study, rhizosphere networks for wild oat were more complex than those in surrounding soils, indicating the rhizosphere has a greater potential for interactions and niche-sharing^56^. Roots might promote the development of niches populated by dominant taxa, which would concurrently yield greater interactions, greater co-variations due to shared niches, and overall result in more complex co-occurrence patterns over time. Conversely, the complex microbial interactions including cooperative or syntrophic interactions among PGPRs might also be benefit for plant growth and health. Microorganisms can communicate with each other through various signal molecules^57^. Specially, rhizosphere microorganisms are more competent at producing signal molecules^58^, which might enhance the microbial feedback with plants.

## Conclusion

Overall, our results showed that soil depth had a highly significant effect on the bacterial communities. Both switchgrass cultivation and fallow soils exhibited the significant negative vertical spatial decay of bacterial similarity relationships. Some dominated taxa regularly changed across soil profiles. However, five-year switchgrass cultivations did not significantly change the structure of soil bacterial community, but generated more beta-diversity variations across soil depth. Furthermore, the bacterial co-occurrence patterns were substantially influenced by switchgrass cultivation. More connection and closer relationships of bacterial taxa were observed in soils under switchgrass cultivation. In future works, more complete information of microbial taxonomic and functional data should be integrated to better understand of the microbial ecology of the soil profile and their response to long-term switchgrass cultivation.

## Materials

### Study area and soil sampling

The switchgrass experiment was carried out over the period 2011–2015 in an experimental area of Northwest A&F University, located in the Guanzhong plain of Shaanxi Province (Fig. 1). The soil series was a clay loam. Switchgrass (cultivars Cave-in-rock and Sunburst) plots were established in September 2011, where winter wheat was cultivated before. Switchgrass was sown into the plots at a seeding rate of 11.2 kg pure live seed ha^−1^ and fertilized with 56 kg N ha^−1^. The fallow plots were adjacent to the switchgrass plots. Both plots were rain fed and no irrigation. After planting, no weed control and no additional fertilizers were applied. The research plots for switchgrass and fallow were 5 × 6 m and replicated three times.

Soil samples were randomly collected from the field in each switchgrass and fallow plots on October 15, 2015. Soil cores were collected with a core sampler at four depths (0–10, 10–20, 20–30 and 30–60 cm). In total, twenty-four soil samples (two plots × four depths × three replicates) were collected, transported to the laboratory in sterile plastic bags on dry ice, and then stored at −80 °C for microbial analyses.

### DNA extraction and purification

Community DNA was extracted from 0.5 g of soil samples using the MP FastDNA®SPIN Kit for soil (MP Biochemicals, Solon, OH, USA) according to the manufacturer protocol. The V4 hypervariable regions of the 16S rRNA gene was amplified using primers 515 F (5′-GTG CCA GCM GCC GCG GTA A-3′) and 806 R (5′-GGA CTA CHV GGG TWT CTA AT-3′), with the forward primer modified to contain a unique 6 nt barcode at the 5′ end. All PCR reactions were performed with 30 μl system with 15 μL of Phusion® High-Fidelity PCR Master Mix (New England Biolabs), 0.2 μM of forward and reverse primers and about 10 ng template DNA. The thermal cycling conditions as following: initial denaturation at 98 °C for 1 min, followed by 30 cycles of denaturation at 98 °C for 10 s, annealing at 50 °C for 30 s, and extension at 72 °C for 60 s, and an extension step at 72 °C for 5 min after cycling was complete. All samples were amplified in triplicate, and no-template controls were included in all steps of the process. Triplicate PCR amplicons were pooled together and then mixed with the same volume of 1 × loading buffer (containing SYB green). They were detected by electrophoresis in a 2% (w/v) agarose gel. PCR products with bright bands were mixed in equal density ratios and purified with GeneJET Gel Extraction Kit (Thermo Scientific, MA, USA). The purified PCR amplicons were sequenced using the Illumina HiSeq 2500 platform at Novogene Bioinformatics Technology Co., Ltd. (Beijing, China).

### Sequence analysis of the 16S rRNA amplicons

Paired-end reads were merged using FLASH (V1.2.7, http://ccb.jhu.edu/software/FLASH/), and filtered according to the literature^59^. The acquired sequences were chimera detected and removed using USEARCH software based on the UCHIME algorithm^60^. The sequences were assigned to each sample with the unique barcodes. Sequence analysis was performed by the UPARSE software package using the UPARSE-OTU and UPARSE-OTUref algorithms. Operational taxonomic units (OTUs) were clustered at the 97% similarity level^61^. Singletons were removed from downstream analyses. The representative sequences for each OTU were assigned to their taxonomic group using the RDP classifier at an 80% confidence threshold^59^.

### Data analyses

Alpha and beta diversity were calculated based on 29126 reads per sample (minimum number of sequences required to normalize the differences in sequencing depth) using QIIME (http://qiime.org/index.html), with multiple indices (observed species and Shannon-Wiener index) and the Bray-Curtis distance between samples. Constrained analysis of principal coordinates (CAP) based on Bray-Curtis distance was performed to investigate the relationship between microbial community composition and soil depth under switchgrass and fallow plots. Canonical discriminant analysis (CDA) was used to identify the taxa associated with different soil layers based on genera with relative abundance levels >0.5%. ANOSIM^62^ and permutational multivariate analysis of variance (PERMANOVA)^63^ were performed to determine whether samples from each groups contained significant differences in their species diversity. The vertical spatial decay of microbial similarity was calculated as the linear least-squares regression relationships between soil depth and the microbial similarity (based on 1 – dissimilarity of the Bray-Curtis distance metric).

Network was used to explore co-occurrence patterns of microbial taxa within switchgrass and fallow soils. The genera with relative abundances above 0.05% were selected. A Spearman’s correlation between two genera was considered statistically robust if the Spearman’s correlation coefficient (ρ) was >0.6 and the *P*-value was <0.01^22^. All the robust correlations identified from pairwise comparison of the genera abundance form a correlation network where each node represents one genus, and each edge stands for a strong and significant correlation between the nodes. To describe the topology of the resulting networks, a set of measures (number of nodes and edges, average path length, network diameter, average degree, clustering coefficient and modularity) was calculated using igraph^64^ packages in R environment and networks were visualized using the interactive platform Gephi^65–67^. The betweenness centrality values of each node were estimated. This topological feature indicated the relevance of a node as capable of holding together communicating nodes, were used to define the keystone species^49, 52^.

All statistics analyses were performed in R environment (http://www.r-project.org) unless otherwise indicated.

## Electronic supplementary material

Supplementary materials
