# Supplementary material for: Ecological diversity and co-occurrence patterns of bacterial community through soil profile in response to long-term switchgrass cultivation
Source: Sci Rep. 2017 Jun 15;7:3608. doi: 10.1038/s41598-017-03778-7 (PMC5472595; doi:10.1038/s41598-017-03778-7)

**Supplementary materials for**

**Ecological diversity and co-occurrence patterns of bacterial community through soil profile in response to long-term switchgrass cultivation**

Shubin He^1^, Lixiang Guo^1^, Mengying Niu^1^, Fuhong Miao^2^, Shuo Jiao^3^, Tianming Hu^1^*and Mingxiu Long^1^*

**Table S1** The vertical spatial variations of the dominated bacterial taxa at phylum level in the fallow and switchgrass soils, correlations between relative abundance of these taxa and soil depths were estimated via Pearson coefficient.

| **Phylum** | **Fallow** | |  | **Switchgrass** | |
| --- | --- | --- | --- | --- | --- |
|  | Pearson coefficient | *P* value |  | Pearson coefficient | *P* value |
| Proteobacteria | -0.057 | 0.861 |  | 0.356 | 0.256 |
| Acidobacteria | -0.254 | 0.425 |  | **-0.761** | **0.004** |
| Bacteroidetes | 0.047 | 0.884 |  | 0.373 | 0.232 |
| Firmicutes | 0.303 | 0.338 |  | **0.655** | **0.021** |
| Gemmatimonadetes | -0.533 | 0.075 |  | -0.391 | 0.209 |
| Planctomycetes | -0.066 | 0.838 |  | -0.469 | 0.124 |
| Actinobacteria | 0.163 | 0.613 |  | 0.285 | 0.369 |
| Verrucomicrobia | -0.371 | 0.236 |  | **-0.687** | **0.014** |
| Chloroflexi | 0.120 | 0.710 |  | 0.289 | 0.362 |
| Nitrospirae | -0.085 | 0.792 |  | -0.384 | 0.218 |
| Crenarchaeota | **-0.708** | **0.010** |  | -0.271 | 0.395 |
| WS3 | 0.401 | 0.196 |  | -0.331 | 0.294 |
| Cyanobacteria | 0.477 | 0.117 |  | **0.552** | **0.063** |
| Armatimonadetes | -0.417 | 0.177 |  | **-0.738** | **0.006** |
| Chlorobi | 0.136 | 0.673 |  | 0.027 | 0.933 |

**Fig. S1** Relative abundances of bacterial classes at 0-60 cm depth in switchgrass and fallow soils.


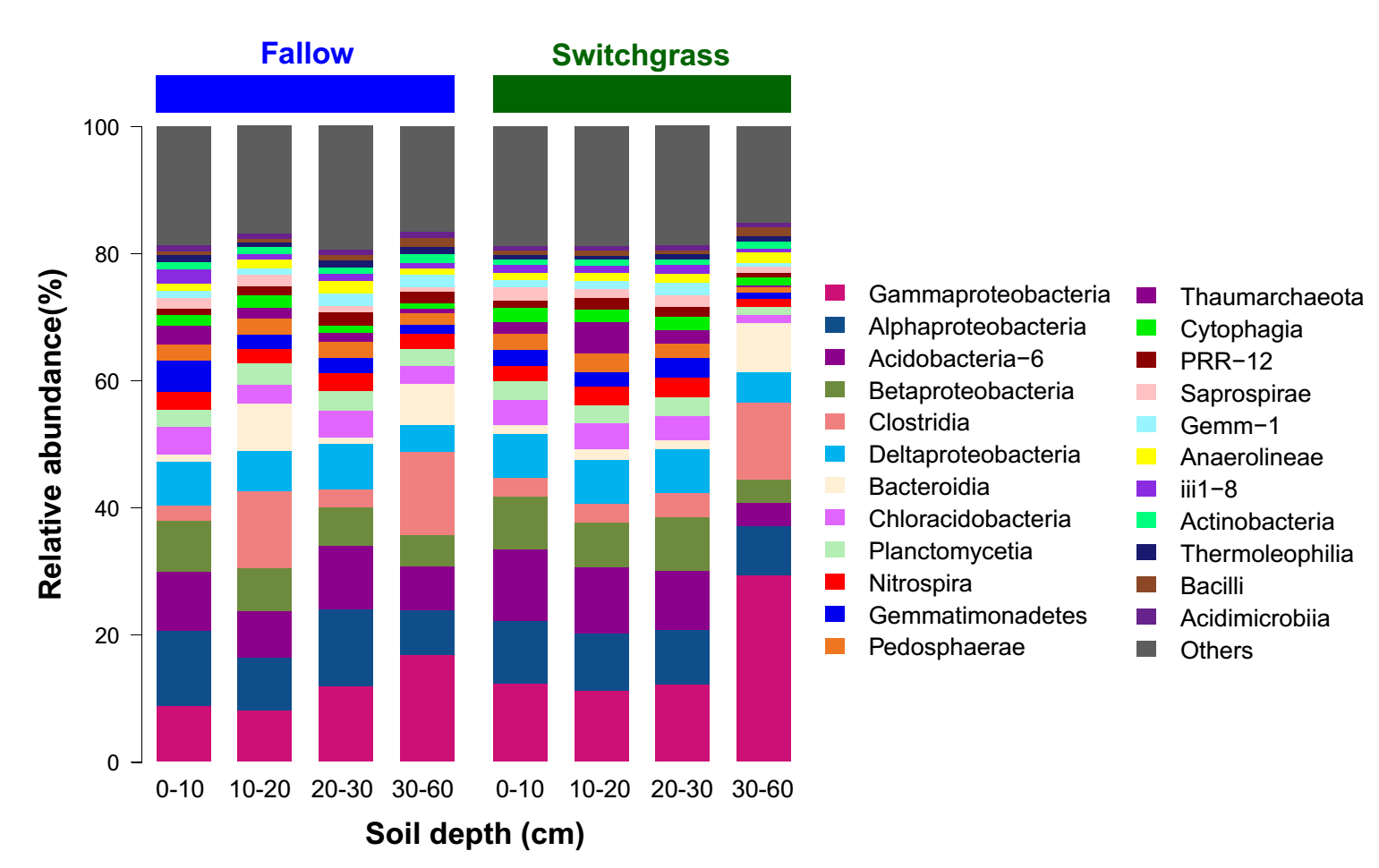


**Fig. S2** The difference of observed OTUs richness between switchgrass cultivation soils and fallow soils. The difference were significant tested by Wilcoxon rank-sum test (*P*<0.05).


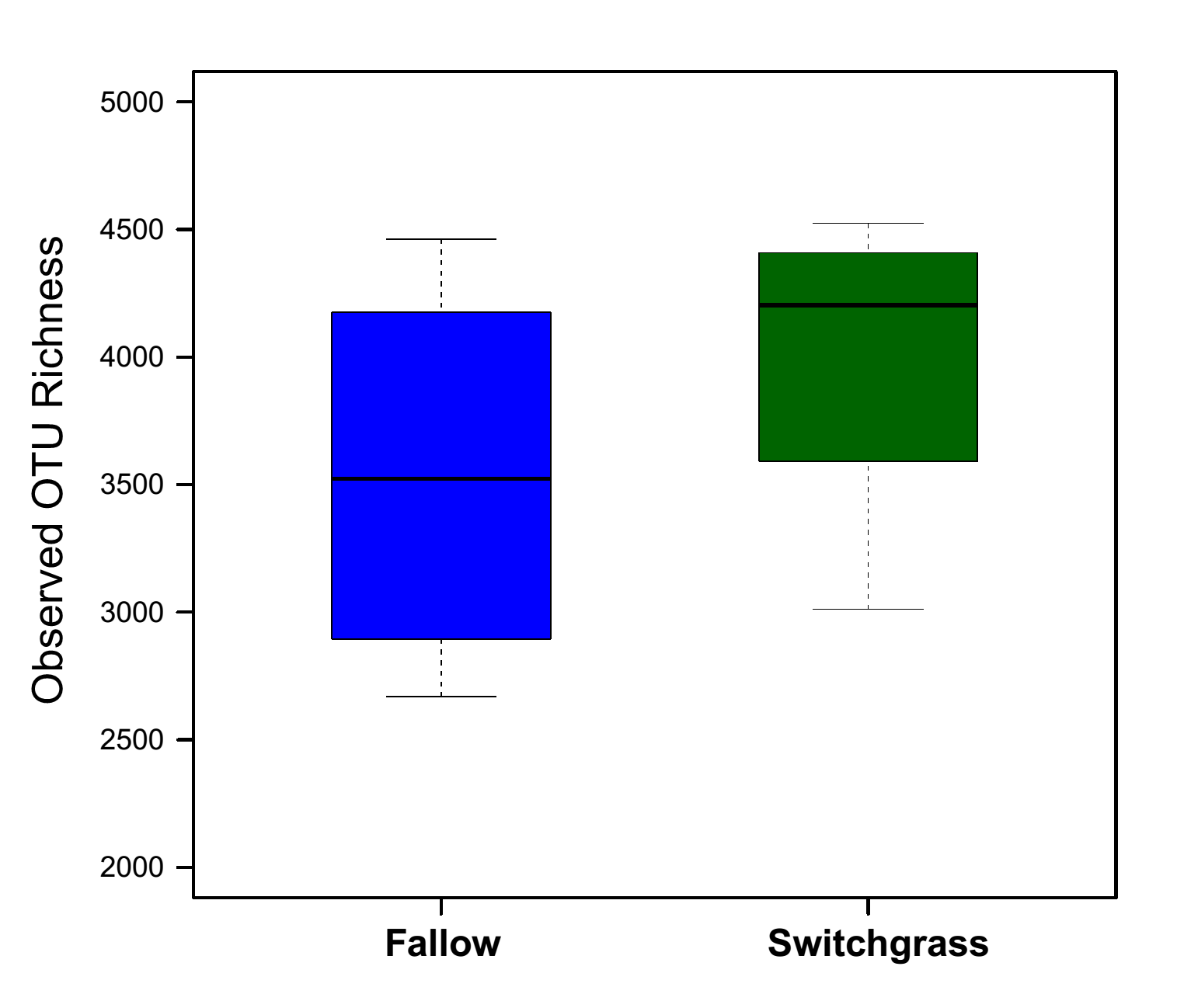


**Fig. S3** Bacterial taxa that had significantly higher relative abundance in switchgrass cultivation soils than fallow soils. The significant differences were examined by Wilcoxon rank-sum test (*P*<0.05) based on the top 1000 most abundant OTUs, which were visualized using the heatmap. Each row in the heatmap has been standardized to have a mean of zero and a standard deviation of one. The intensity of the color in the heatmap is proportional to the standardized relative abundance of the taxa.


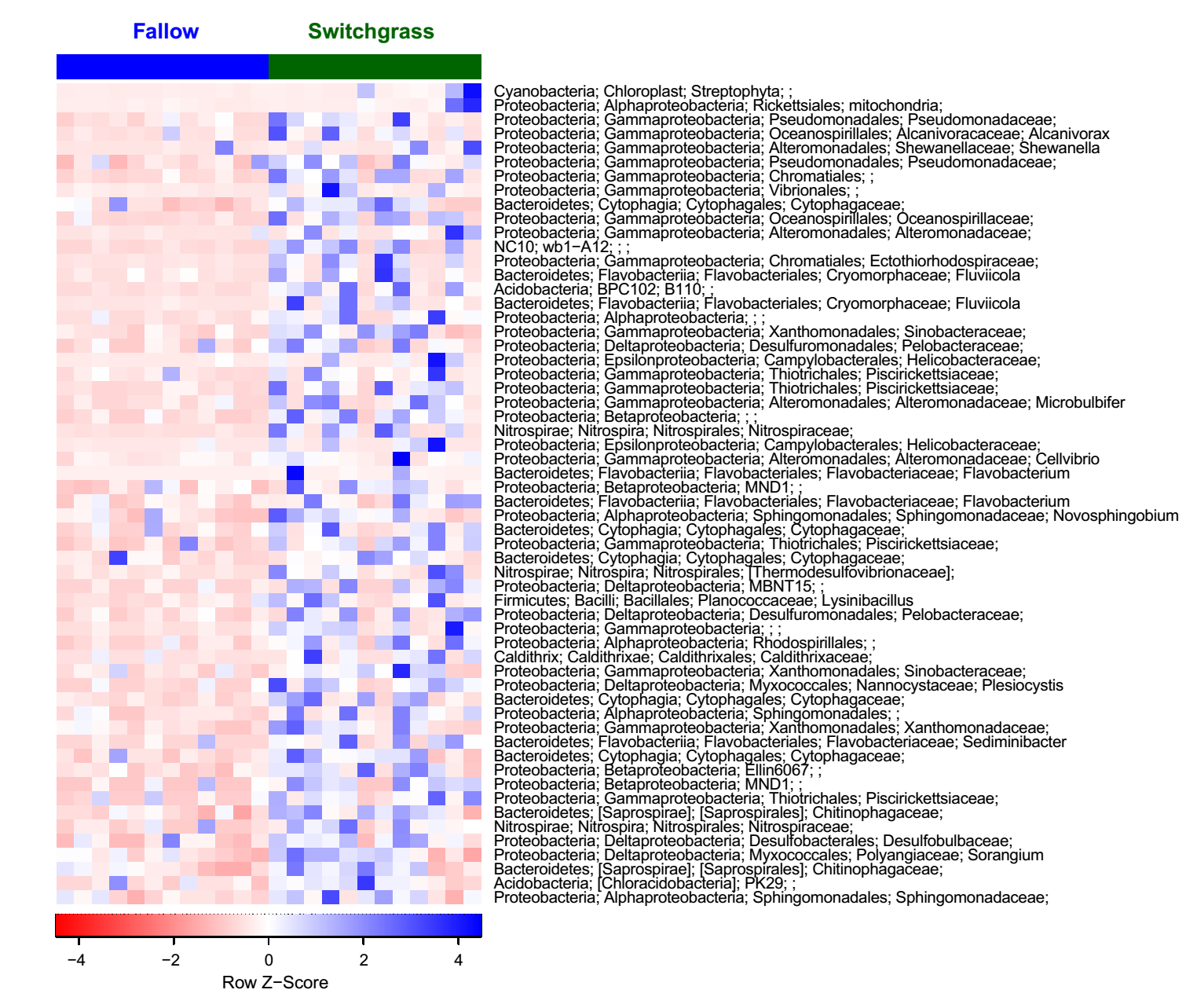


**Fig. S4** Bacterial taxa that had significantly higher relative abundance in fallow soils than switchgrass cultivation soils. The significant differences were examined by Wilcoxon rank-sum test (*P*<0.05) based on the top 1000 most abundant OTUs, which were visualized using the heatmap. Each row in the heatmap has been standardized to have a mean of zero and a standard deviation of one. The intensity of the color in the heatmap is proportional to the standardized relative abundance of the taxa.


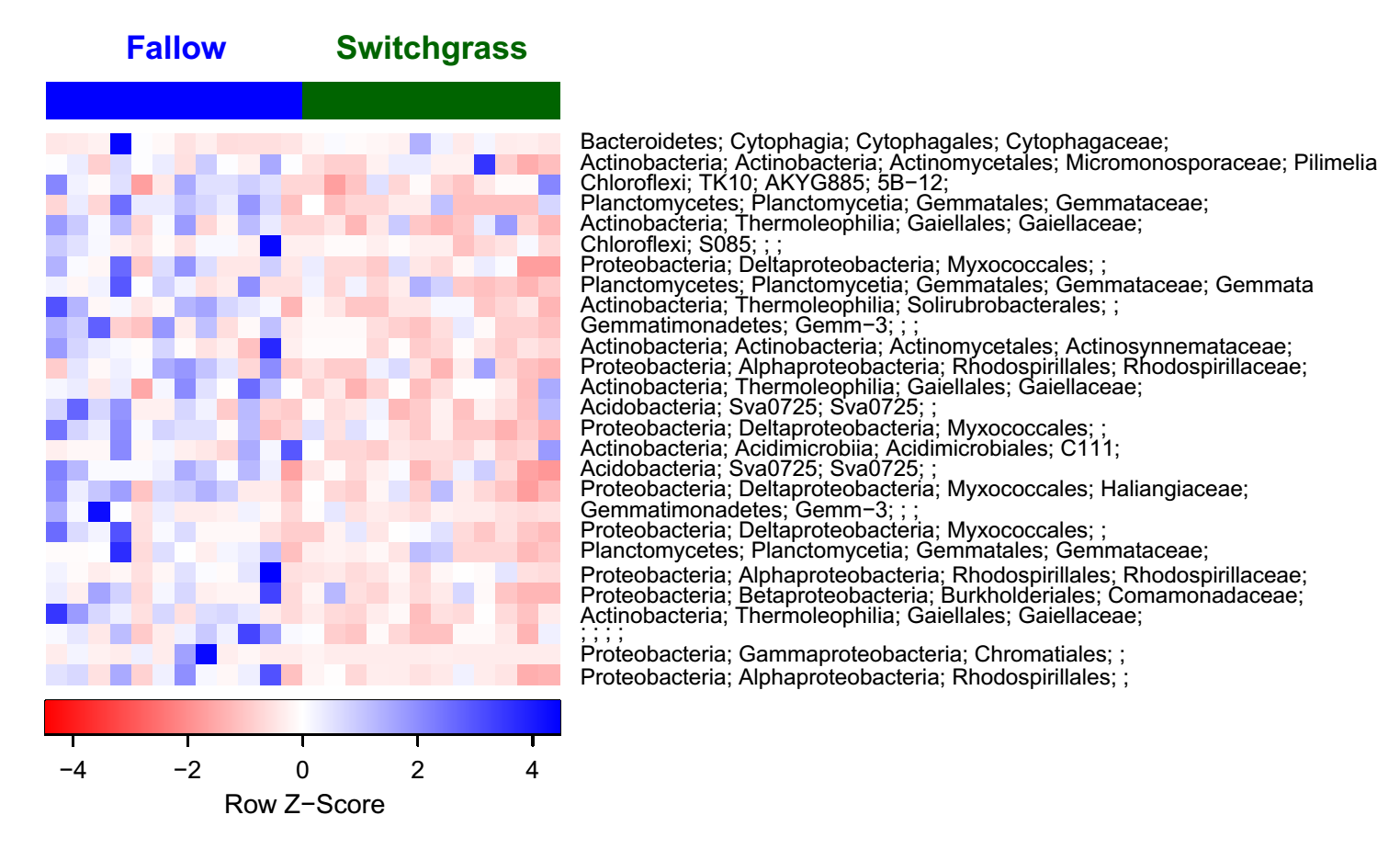


**Fig. S5** The difference of betweenness centrality between switchgrass and fallow network. The values were significantly different estimated via Wilcoxon Rank Sum tests (*P* < 0.05).


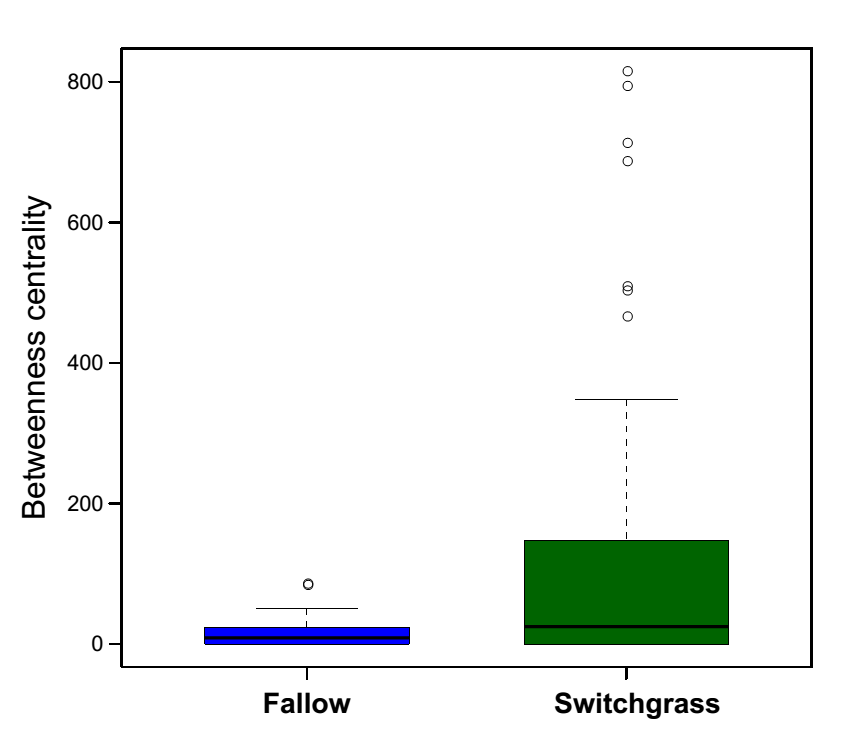


**Fig. S6** The difference of soil organic carbon between switchgrass cultivation and fallow soils through soil layers. The values of each layer were significantly different between switchgrass cultivation and fallow soils, estimated via Wilcoxon Rank Sum tests (*P* < 0.05).


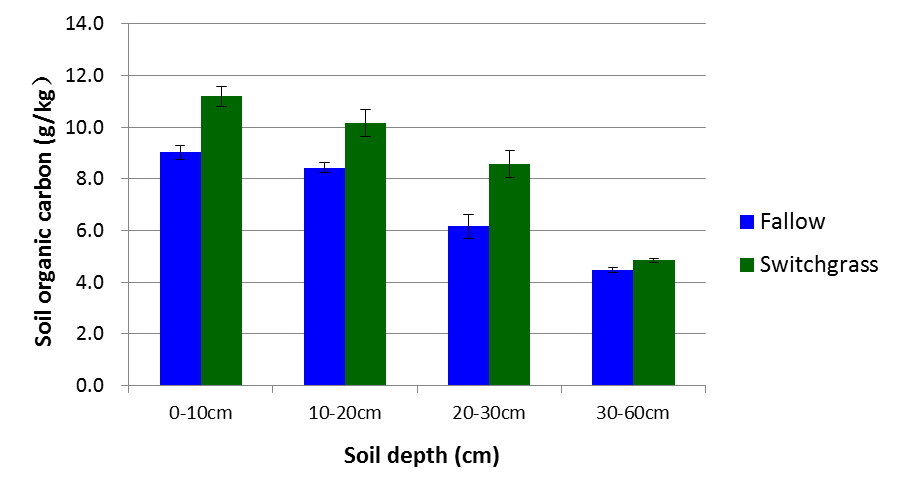

Supplement: Supplementary file 1 — Supplementary materials [file 41598_2017_3778_MOESM1_ESM.doc]
